# Cancer Biomarkers Discovery of Methylation Modification With Direct High-Throughput Nanopore Sequencing

**DOI:** 10.3389/fgene.2021.672804

**Published:** 2021-05-26

**Authors:** Junjie Zhang, Shuilian Xie, Jingxiang Xu, Hui Liu, Shaogui Wan

**Affiliations:** ^1^Center for Molecular Pathology, Department of Basic Medicine, Gannan Medical University, Ganzhou, China; ^2^Institute of Hepatology, The Affiliated Fifth People’s Hospital of Ganzhou, Gannan Medical University, Ganzhou, China

**Keywords:** nanopore sequencing, cancer biomarker, Cas9 enrichment, DNA methylation, RNA methylation

## Abstract

Cancer is a complex disease, driven by a combination of genetic and epigenetic alterations. DNA and RNA methylation modifications are the most common epigenetic events that play critical roles in cancer development and progression. Bisulfite converted sequencing is a widely used technique to detect base modifications in DNA methylation, but its main drawbacks lie in DNA degradation, lack of specificity, or short reads with low sequence diversity. The nanopore sequencing technology can directly detect base modifications in native DNA as well as RNA without harsh chemical treatment, compared to bisulfite sequencing. Furthermore, CRISPR/Cas9-targeted enrichment nanopore sequencing techniques are straightforward and cost-effective when targeting genomic regions are of interest. In this review, we mainly focus on DNA and RNA methylation modification detection in cancer with the current nanopore sequencing approaches. We also present the respective strengths, weaknesses of nanopore sequencing techniques, and their future translational applications in identification of epigenetic biomarkers for cancer detection and prognosis.

## Introduction

Epigenetic alternations are important to different types of human cancers and are now known to link genetic changes to drive the initiation and progress of cancers. These changes can be observed as abnormal patterns of DNA methylation, disrupted patterns of histone modifications, and changes in chromatin components. More than 17 types of modification in DNA and 160 post-transcriptional modifications in RNA have been found, respectively (Zhao et al., 2020), among which the commonest modification type is methylation modification (Jenjaroenpun et al., 2021). Currently, the identification of cancer epigenetic biomarkers has been emerged by means of high-throughput sequencing technologies.

The commonest epigenetic change in cancers is 5-methylcytosine (5mC) DNA modification. For the detection of 5mC in DNA, bisulfite converted sequencing is the most commonly applied method. Although bisulfite-based sequencing remains the gold-standard method for DNA epigenetic analysis, it has a number of limitations and is not easily applicable to resolve multiple cytosine modifications. A significant drawback of these methods is that bisulfite chemical treatment requires a high temperature and different pH conditions. It can cause significant degradation of the original DNA (Tanaka and Okamoto, 2007). Oxford nanopore technologies (ONTs) offer exciting possibilities to study a broad range of epigenetic modifications in cancer, taking the advantages of directly single-molecule sequencing, which is capable of quantitative methylation assessment without bisulfite conversion, as ionic current changes are sensitive to 5mC modified cytosine base (C) (Simpson et al., 2017). Moreover, multiple CpGs in the promoter region of cancer-associated genes or other genomic regions of interest can be captured by nanopore Cas9-targeted sequencing (nCATS) (Gilpatrick et al., 2020). In this review, we introduce recent nanopore sequencing techniques in detection of DNA and RNA epigenetic modifications in cancer. We also summarize the advantages and disadvantages of nanopore sequencing and their future translational applications into the discovery of putative epigenetic biomarkers for diagnosis and prognosis prediction of human cancers.

## The Formation of DNA/RNA Modifications

DNA modifications have crucial roles in several biological processes, including cancer (Koch et al., 2018) and aging (Unnikrishnan et al., 2019). 5mC occurs predominantly at CpG dinucleotides in DNA. The patterns of genomic methylation are performed and maintained by three enzymes, DNA methyltransferase (DNMT) 1, 3A, and 3B (Jin et al., 2011). Additionally, 5-hydroxymethyl (5hmC) is generated via ten-eleven translocation (TET)–mediated oxidation of 5mC, which serve as an active intermediate of DNA demethylation in mammals (Kohli and Zhang, 2013). RNA modifications also play critical roles in diverse biological processes, including development and cancer (Frye et al., 2018; Delaunay and Frye, 2019). N6-methyladenine (m6A) is the most abundant mRNA modification in eukaryotic cells. The majority of m6A on mRNA is installed by the “writer” complex consisting of METTL3, METTL14, and WTAP (Li A. et al., 2017) and can be removed by the “erasers” FTO and ALKBH5 (Jia et al., 2011; Zhang et al., 2015). Readers, such as the YTH family proteins, directly or indirectly recognize the m6A-marked transcripts and affect various aspects of mRNA metabolism (Xiao et al., 2016; Li A. et al., 2017; Roundtree et al., 2017; Shi et al., 2017).

## Current Methods for Detecting DNA and RNA Modifications

Because of the different chemical properties corresponding to every modified type of nucleic acids, the methods for detecting each modification are also extremely diverse (Chen et al., 2020). Here, we primarily cite the most representative modified bases, such as 5mC/5hmC in DNA and m6A in RNA, as examples to illustrate relevant methods and technologies of detection.

At present, the whole-genome bisulfite sequencing (WGBS) (Kernaleguen et al., 2018) mainly uses bisulfite-treated samples for next-generation sequencing (NGS). The principle is that bisulfite does not affect methylated C (5mC and 5hmC), but it can transform normal C into uracil (U), which can be read out as thymine (T) in subsequent polymerase chain reaction (PCR) amplification. Despite WGBS as the predominant method to detect genetic methylation currently, it has many shortcomings including DNA destruction by bisulfite treatment, PCR-induced GC bias, and inability to distinguish between 5mC and 5hmC. Similar detection methods include bisulfite-converted Sanger sequencing (Frommer et al., 1992) and reduced representation bisulfite sequencing (Gu et al., 2011). In order to solve the problems caused by bisulfite treatment, some bisulfite-free methods have emerged in recent years, such as TET-assisted pyridine borane sequencing (Liu Y. et al., 2019) and Enzymatic Methyl-Seq (Vaisvila et al., 2019), which are based on the catalysis of specific enzymes. In the detection of 5hmC, Han et al. (2016) developed 5hmC selective chemical labeling technology (hmC-Seal) by combining chemical synthesis technology with modern biological technology. It opens the door for the research and clinical transformation of 5hmc (Gao et al., 2019; Dong et al., 2020). However, this method is unable to obtain specific 5hmc modification sites at a single-base level. Chemical-assisted C-to-T conversion of 5hmC sequencing (hmC-CATCH) (Zeng et al., 2018) overcomes this difficulty by selectively oxidizing 5hmC, but leaving C and 5mC intact. Over the same period, another new method called APOBEC-coupled epigenetic sequencing (ACE-Seq) (Schutsky et al., 2018), contrary to hmC-CATCH on converted subjects, uses DNA deaminase APOBEC3A (A3A) to specifically remove the amino group of C and 5mC to make them U, whereas 5hmC is still detected as C. Unfortunately, not all the types of modification can be identified by suitable chemical reagents or specific enzymes.

Up to now, the methods of detecting m6A in RNA are various (Helm and Motorin, 2017). The most widely used technology is MeRIP-Seq/m6A-Seq (Dominissini et al., 2012), which is based on the reaction of antibody (m6A antibody) and antigen (m6A) to enrich RNA fragments with abundant m6A for sequencing. But because of the non-specific binding of m6A antibody, the false-positive rate is high, and its resolution can only reach about 100 nt RNA. For this reason, many technologies, such as miCLIP (Linder et al., 2015) and m6A-REF-seq (Zhang et al., 2019), are constantly improved to achieve the accuracy of single-base resolution.

It is worth noting that the methods listed previously basically require one of the three complex pre-treatments, namely, chemical conversion, enzyme recognition, and antibody enrichment (Chen et al., 2020). Nanopore sequencing technology, one of the representatives of the third-generation sequencing technology, can exceed most of the shortcomings in the current methods. It directly sequences DNA or RNA modification without pre-treatment, is not bound by the length of sequencing compared with NGS, and hence has been widely used (Rand et al., 2017; Simpson et al., 2017; Garalde et al., 2018; Liu H. et al., 2019; Liu Q. et al., 2019).

## Detection of DNA Modifications by Nanopore Sequencing

The importance of DNA modifications such as 5mC and m6A on gene expression and function has become increasingly apparent. For example, 5mC has been related to many human diseases, such as neurological disorders (Weng et al., 2013) and cancer (Simpson et al., 2017), and may serve as a potential diagnostic and prognostic biomarker. The cancer cell genome undergoes dramatic shifts in the pattern of genomic methylation, including genome-wide hypomethylation in conjunction with local areas of hypermethylation. Aberrant hypomethylation causes the expression of certain genes, such as oncogenes, whereas hypermethylation causes the inhibition of tumor suppressor genes (Feinberg and Vogelstein, 1983; Esteller et al., 2001). A major benefit of nanopore sequencing is the ability to directly sequence DNA molecules to identify base modifications with high accuracy owing to the characteristic signatures caused by the modified base as it translocates through the nanopore (Rand et al., 2017; Simpson et al., 2017; Gigante et al., 2019). Many studies have been carried out to detect DNA modifications in cancer with nanopore sequencing (Table 1). For example, Simpson et al. (2017), developed a nanopore-based detection method, Nanopolish, which is based on the hidden Markov model (HMM) and can directly distinguish 5mC from unmethylated cytosine without any chemical treatment. They applied their method to two human breast cell lines, MCF10A (a non-tumorigenic epithelial cell line) and MDA-MB-231 (an aggressive metastatic cancer cell line). They precisely identified a hypermethylated island region at chromosome 9 when comparing methylation calls in cancer cells (MDA-MB-231) to those in normal cells (MCF10A) utilizing both the nanopore and bisulfite sequencing data. However, this approach required a training set from completely CpG-methylated DNA, which reduced the ability to detect heterogeneous methylation within a region. The request for more comprehensive training sets would probably address this issue. In another report by Euskirchen et al. (2017) they employed MinION platform to simultaneously detect structural variants, point mutations, and DNA methylation profile in brain tumors. In order to call 5mC from various basecalling groups, they attempted to modify the original implement of Nanopolish 0.6.0. They first made a comparison between methylation events in CpG sites detected by nanopore sequencing and matched methylome microarrays and then observed good relationship between single-read methylation status of a given CpG site and its corresponding beta value determined by microarray analysis. They then compared the classification of tumor by using copy number profile alone, DNA methylation only, or both modalities together; they found all samples were accurately classified using the joint approach. In addition, they observed, although with a low depth of genome coverage, the methylation data are sufficient to subtype gliomas into IDH-mutant versus wild-type samples and also able to rapidly distinguish cancer entities from different tissue origins. Moreover, one recently published article has demonstrated it is possible to accurately assess methylation of transposable elements (TEs) in cancer by using long-read nanopore sequencing (Ewing et al., 2020). They utilized ONT PromethION platform to sequencing clinical samples including paired tumor/non-tumor liver tissue from a hepatocellular carcinoma patient. They obtained aberrant and allele-specific TE methylation in normal tissue and successfully identified pronounced demethylation of young long interspersed element 1 (LINE-1) retrotransposons in cancer, often distinct to the adjacent genome and other young TEs. Furthermore, nanopore sequencing is also capable to simultaneously study native CpG methylation and chromatin accessibility (Lee et al., 2020; Shipony et al., 2020). The degree of packing of the DNA in the chromatin has an effect on the gene expression because it controls access to the factors that regulate gene transcription. Recently, Lee et al. (2020) have developed nanopore sequencing of nucleosome occupancy and methylome (NanoNOMe), an extension of NOMe-seq (Kelly et al., 2012), where endogenous CpG methylation with exogenous GpC modifications at accessible sites were labeled in mammalian cells. They applied this method to a breast cancer model to evaluate differential methylation and accessibility between cancer and normal cells. They found the cancer cells had higher numbers of hypomethylated differentially methylated regions (DMRs) than hypermethylated DMRs, which suggested global hypomethylation in breast cancer cells. Notably, they also observed the upregulation of *ZNF14* gene occurred concomitant with increased epigenetic activity (unmethylated and accessible) and subnucleosomal footprints near transcription start sites (TSSs) in the cancer cells as compared to normal cells. Nanopore sequencing is also capable to analyze regions of differential methylation between parental alleles, which results in parent-of-origin effects on gene expression. These so-called imprinted regions exhibit differential methylation of CpG sites, and the long reads make it possible to accurately assess the haplotype of each read (“phasing”) (Gigante et al., 2019).

**TABLE 1:** Detection of methylation modification by nanopore sequencing.

**Biomarker**	**Sample**	**Alternation**	**Targeted region**	**Sequencing technology**	**Bioinformatic tool**	**References**
5mC	Two human breast cell lines (MCF10A and MDA-MB-231)	Hypermethylated CpG island in breast cancer cells	Chromosome 9:63,817,919–63,818,178	Nanopore sequencing on MinION; bisulfite-converted sequencing on Illumina MiSeq (for validation)	bsseq package for region-specific analysis of samples from cancer and normal cells; Bismark for the bisulfite-based sequencing data analysis; nanopolish with hidden Markov models (HMMs) used to analyze nanopore sequencing data	Simpson et al., 2017
5mC	Primary brain tumor tissues and tumors metastasizing to the brain	Global hypermethylation of CpG island in IDH-mutant brain tumors	Global DNA profile	Nanopore whole genome sequencing on MinION; NGS exome sequencing, Sanger sequencing, SNP array, and/or genome-wide methylation microarray used for sequencing all tumor samples previously	Albacore 1.1.0 for basecalling; BWA MEM 0.7.12 with the “-x ont2d” option for aligning DNA sequences to the hg19 human reference genome; nanopolish with algorithm based on a hidden Markov model for methylation analysis	Euskirchen et al., 2017
5mC	Hepatocellular carcinoma tissues	Significant tumor-specific long interspersed element 1 (LINE-1) transposon demethylation in cancer tissues	LINE-1	Nanopore whole-genome sequencing on PromethION	Transposons from long DNA reads (TLDR) used to generate element-specific methylation profiles of non-reference transposable element (TE) insertions; nanopolish version 0.11.0 for per-CpG methylation calling	Ewing et al., 2020
5mC	Two breast cancer cell lines (MCF-7 and MDA-MB-231)	Global hypomethylation in breast cancer subtypes; and specific genes with hypermethylation	Genes transcription factor binding sites and gene promoter regions, especially on CTCF-binding sites; transcription start sites (TSS) of ER, PR, HER2 and ZNF714 receptors	Nanopore whole-genome sequencing on MinION, GridION, or PromethION; WGBS on MiSeq for validation of DNA methylation	GUPPY v.3.0.3 for converting raw current signals to DNA sequences; NGMLR v.0.2.8 for aligning DNA sequences to hg38 human reference genome; nanopolish v.0.11.1 for generating GpC methylation model	Lee et al., 2020
5mC	Three breast cell lines (MCF-10A, MCF-7 and MDA-MB-231), mouse xenograft, primary tumor tissues	Hypomethylation of KRT19 gene in tumorigenic MCF-7 and MDA-MB-231 cell lines and in primary tumor tissues	KRT19, SLC12A4, GSTP1, TPM2 and GPX1 regions	Nanopore Cas9-targeted sequencing (nCATS) on MinION or GridION	GUPPY (v.3.0.3) for basecalling to generate FASTQ sequencing reads from the electrical data; minimap2 (v.2.17) for aligning the reads to the human reference genome (Hg38); nanopolish (v.0.11.1) for CpG methylation calling on nanopore data	Gilpatrick et al., 2020
5mC	Glioblastoma (GBM) tissues; four GBM cell lines (U87, U251,T98G, and LN18)	Hypomethylation in TMZ-resistant cell lines high MGMT expression	O6-methylguanine-DNA methyltransferase (MGMT) promoter region and MGMT CpGs across the proximal promoter region, the entirety of exon 1, and a portion of intron 1	Nanopore Cas9-targeted sequencing (nCATS) on MinION	Nanopolish v 0.11.0 (17) using the reads (FASTQ files), aligned reads (BAM files), and raw signals (FAST5 files) for each sample to perform CpG methylation (5mC) calling	Wongsurawat et al., 2020
5mC	Papillary (TPC-1, BCPAP) and follicular thyroid cancer cell lines (FTC-133, FTC-238,and WRO) with telomerase reverse transcriptase (TERT) mutation	Hypomethylation on the mutant TERT gene	Transcription start site (TSS), TERT mutation site, and transcription factors MYC binding site on the mutant TERT allele compared to the wildtype allele	Nanopore Cas9-targeted sequencing (nCATS) on GridION	GUPPY algorithm for performing Base calling to generate FASTQ reads; Minimap2 for aligning the resulting reads to the human genome (hg19); nanopolish for conducting CpG methylation calling	McKelvey et al., 2020
m6A	Multiple cancer cell lines, such as HEK293T, HepG2, HCT116, MCF7, A549, and K562	Hypermethylation at the significantly differentially modified sites for each cancer cell line compared to Mettl3-knockout HEK293T cells	The DRACH motif; global levels (high levels of variation across positions and samples)	Nanopore Direct RNA-Sequencing; m6A-Crosslinking-Exonuclease-sequencing (m6ACE-seq) used as m6A reference	xPore for the analysis of differential RNA modifications from direct RNA-Sequencing data	Pratanwanich et al., 2020
m6A	Human lung cancer cell lines H460	Hypermethylation on DARCH motif regions or globally	The DRACH/RRACH motif; the consensus DRACH-like motif; global levels	Nanopore Direct RNA sequencing on MinION	ELIGOS based on various types of synthetic modified RNA and applied to rRNA and mRNA to compare the error profile between native RNA sequences obtained with dRNA-seq and a reference	Jenjaroenpun et al., 2021

Several bioinformatics approaches have been developed to call DNA modifications on the basis of the electric signal obtained during the sequencing run: Nanopolish (Loman et al., 2015; Simpson et al., 2017), signalAlign (Rand et al., 2017), DeepSignal (Ni et al., 2019), mCaller (McIntyre et al., 2019), DeepMod (Liu Q. et al., 2019), and Tombo (Stoiber et al., 2017). Detailed discussion of these methods is available in Xu and Seki (2020). These tools have been used to uncover methylation states in previously inaccessible genomic regions, such as the X chromosome centromere (Miga et al., 2020), as well as genes implicated in cancer (Lee et al., 2020), leading to new biological insights into, particularly, the methylation status on the X chromosome.

## Detection of RNA Modification by Nanopore Sequencing

Methods for detection of RNA modifications, including antibody immunoprecipitation (e.g., MeRIP-seq, miCLIP) (Helm and Motorin, 2017; Li X. et al., 2017) and chemical-based modification, required to convert RNA to complementary DNA (cDNA). However, cDNA-based methods through reverse transcription or amplification might introduce bias (Garalde et al., 2018). These issues can be exacerbated with traditional short-read sequencing technologies, which are known to exhibit GC bias. Additionally, these technologies rely on available antibodies or known enzymes are often unable to detect the underlying RNA molecule that is modified, such base modifications are known to have a role in modulating the activity and stability of RNA and are therefore of increasing interest to researchers. Furthermore, the requirement for complex protocols makes these methods difficult to build a large-scale application.

To address these limitations, direct RNA sequencing platform provided by ONT has emerged as an ideal alternative technology, which has the potential to detect sites of modification in native RNA molecule (Garalde et al., 2018). Direct RNA nanopore sequencing has been used to analyze m6A in yeast (Garalde et al., 2018; Liu H. et al., 2019), *Arabidopsis* (Parker et al., 2020), RNA virus genomes (Kim et al., 2020), and human cells (Leger et al., 2019; Workman et al., 2019; Lorenz et al., 2020; Pratanwanich et al., 2020; Jenjaroenpun et al., 2021). For example, Workman et al. (2019) conducted direct RNA-seq analysis of RNA from a human cell line GM12878, *in vitro* transcribed RNA from cDNA from the same cell line, and synthetic RNA. These authors focus on the m6A methyltransferase-binding motif and obtained current differences for the motif. They subsequently validated the differences in signals utilizing data from the synthetic RNA. Interestingly, m6A-modified motifs in a group of genes were detected by using the current difference. Recently, a computational method known as xPore developed by Pratanwanich et al. (2020) enabled differential RNA modifications from direct RNA sequencing data to be retained (Table 1). They tested this method on direct RNA sequencing data across six genetically distinct human cell lines covering HEK293T-KO cells, liver cancer cells (HEPG2), colon cancer cells (HCT116), breast cancer cells (MCF7), lung adenocarcinoma cells (A549), and leukemia cells (K562). When compared to the HEK293T-KO cells, between 800 and 2,000 differentially modified sites were identified for all five cancer cell lines; the vast majority of sites conformed to m6A DRACH motif. Their findings showed that RNA modifications can be observed across conditions, even when samples have a diverse genetic background. Also in the same study, the dynamics of m6A were investigated across the different tissues represented by the cell lines. Intriguingly, they found that many m6A sites are preserved across cell lines with most positions being shared. Moreover, one of the advantages of xPore is that it is suited for detecting m6A with direct RNA-seq data from clinical cancer samples even with limited RNA (2.5 ug), opening new avenues for larger-scale analysis of clinical patient data. Additionally, Jenjaroenpun et al. (2021) employed native RNA sequencing on lung cancer cell line H460 and detected m6A in the RNA using ELIGOS. They also applied ELIGOS to analyze a published reference native RNA sequencing dataset of the human cell line CEPH1463 (Workman et al., 2019). Comparison of the identified m6A sites between H460 and CEPH1463 cells revealed that most of the m6A modification sites in lung cells were highly enriched in CEPH1463 cells. Even though lung cell datasets have relative shallow sequencing depths, they observed a number of m6A sites that were identified in the lung cells and not detected in the CEPH1463 cell, suggesting that the cell type–specific regulation of m6A RNA modification. Finally, they evaluated the ELGOS results of m6A with other published methods; this analysis showed that ELIGOS accurately detected the m6A position at single-base resolution with high concordance to miCLIP (Linder et al., 2015) and UV-CLIP (Ke et al., 2015) methods. Although still in its infancy, direct RNA sequencing has the potential to detect RNA base modifications and therefore has high potential to update current knowledge of epitranscriptome in cancer.

## Targeted Enrichment for Nanopore Sequencing

Targeted sequencing has proven to be economical for obtaining sequencing data with high coverage and quality for specific genomic regions. Deep sequencing coverage is important for interrogation of heterogeneous methylation profile across clinical samples. However, most targeted enrichment sequencing methods require amplification that can have negative effect on subsequent analysis. For instance, the process of amplification removes all information on base modifications present in native DNA, thereby losing a potentially informative source of variation. In addition, some genomic regions such as those with typically high GC content and repetitive sequences are recalcitrant to faithful amplification. Yet, a group of human genetic disorders is caused by repeat expansions. In order to overcome these challenges, researchers are now investigating the potential of CRISPR/Cas9 techniques to enrich for defined regions of interest DNA fragment (Table 1). CRISPR-Cas9 in combination with long-read sequencing is being developed for nanopore sequencing (Gilpatrick et al., 2020), as well as PacBio sequencing (Tsai et al., 2017). Such methods have shown promise for achieving the higher sequence coverage needed for accurate base modification detection, which has led to the discovery of methylation profile differences in diseased and healthy individuals and has been used to identify novel hypomethylated regions in the genome.

**FIGURE 1:**
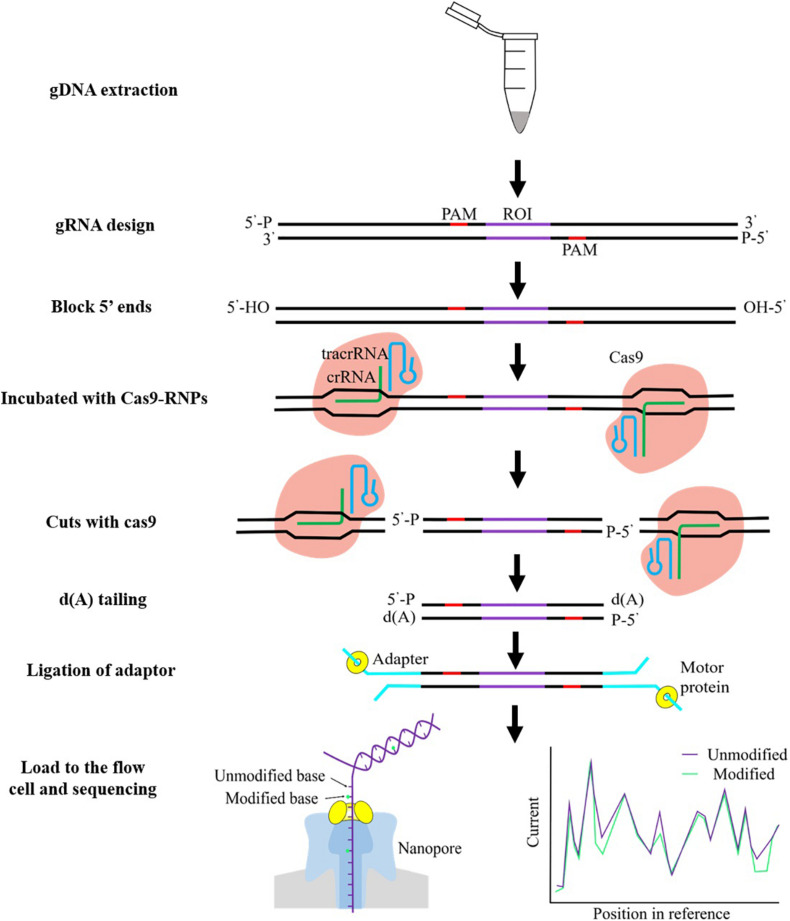
Schematic overview of Cas9 enrichment workflow. Genomic DNA (gDNA) is first extracted from cells or tissue, guide RNA (gRNA) designed, and DNA 5′ ends are dephosphorylated, and then Cas9 ribonucleoprotein complexes (Cas9 RNPs) were used for cleaving the region of interest (ROI). An adenine (A)-tail is rapidly added to the 3′ ends of cut DNA fragments. ONT adapters are ligated to cut end around the ROI. The sequencing library is carefully cleaned to remove excess adapters using AMPure XP beads. The prepared library is loading onto the flow cell for sequencing. DNA modifications can be detected by nanopore sequencing. As the single-stranded DNA moves through the pore, it causes disruptions to the ionic current in a sequence-dependent manner, generating a readout known as a “squiggle.” The current signal highlighted with green lines indicates small changes due to the methylated cytosine.

For example, Gilpatrick et al. (2020) implemented an amplification-free method, termed nanopore nCATS, utilizing the CRISPR-Cas9 system to target cleavage of a region of interest, followed by enrichment and long-read sequencing based on the nanopore sequencing. The schematic overview of nCATS proceedings is shown in Figure 1. They demonstrated the capability of nCATS to assess the methylation profiles for a selection of target genes including *KRT19*, *SLC12A4*, *GSTP1*, *TPM2*, and *GPX1* in three breast cell lines (MCF-10A, MCF-7, and MDA-MB-231). Among these loci, the nanopore-derived methylation patterns were compared against previously released WGBS data in GM12878 (Dunham et al., 2012). They observed a high positive correlation (Pearson *r* = 0.81) by directly comparing per-CpG methylation between nCATS quantitative methylation and WGBS at each locus. Additionally, they explored this approach to examine regions with differential methylation using data from breast cell lines, and then they identified one gene, the keratin family member gene *KRT19*, which showed differential methylation in breast cell lines. As previously reported, the expression of *KRT19* is especially high in breast cancer, and *KRT19* mRNA has been a suitable marker for identifying micrometastasis of breast cancer to lymph nodes and used for circulating tumor cell detection (Wang et al., 2019). Crucially, they found that *KRT19* maintains substantially methylated in the non-tumorigenic MCF-10-A cell line; conversely, *KRT19* presents hypomethylated in the MCF-7 and MDA-MB-231 breast cancer lines, associated with an obtained increased expression for *KRT19* in the transformed cell lines. In a more recent study, McKelvey et al. (2020) investigated on human DNA from thyroid cancer cell lines where they targeted the human *TERT* gene (*hTERT*), which encodes a core protein component of the telomerase complex. Telomerase that acts to maintain the telomeric sequence to chromosomal ends is repressed in almost all somatic cells, although it is commonly expressed in the vast majority of cancer cells (Yi et al., 2001). It is already known that telomerase activity closely correlated with methylation of the *hTERT* promoter (Castelo-Branco et al., 2013). The repetitive nature and high GC content of the *hTERT* gene region make it difficult to analyze using conventional PCR amplicons. Applying the CRISPR/Cas9 method on the thyroid cancer cell line, they enriched the targeted 2.4-kb region with 50-fold increase in coverage. Following analysis of the nanopore sequencing data allowed detection of the epigenetic modification 5mC with high concordance to previously published Illumina bisulfite sequencing of the *hTERT* promoter (Avin et al., 2019). They demonstrated that DNA methylation estimated by nCATS exhibited significant drop in methylation surrounding TSS and higher levels of methylation in the gene body of mutant *hTERT* allele in heterozygous TPC-1 thyroid cancer cell line.

Moreover, the study by Wongsurawat et al. (2020) employed the nCATS to simultaneously evaluate dehydrogenase (*IDH*) gene mutation status and the methylation level of O6-methylguanine-DNA methyltransferase (*MGMT*) promoter in four human glioblastoma (GBM) cell lines and eight fresh human brain tumor samples. It has been known that the *IDH* mutation and *MGMT* promoter methylation status are commonly used as prognostic makers in patients with GBM (Weller et al., 2017). They demonstrated the use of nCATS to accurately detect the *IDH1* and *IDH2* mutations within 36 h, and the assessment of *IDH* mutational status was in agreement with Sanger and Illumina sequencing data. Using a methylated and unmethylated DNA standard, nCATS was able to provide high resolution of *MGMT* methylation pattern along the entire promoter region, exon 1, and a portion of intron 1 in both samples that was comparable to results generated with pyrosequencing assays. For *MGMT* methylation, they also applied nCATS to four well-characterized GBM cell lines and clinical samples and found that there was a significant positive correlation between the percent methylation of these four cell lines assayed by nCATS with the percent methylation returned by pyrosequencing. These MassARRAY^®^ results they obtained also showed a similar trend with nCATS results over the same CpG sites. They further determined the relationship between the methylation level and *MGMT* expression for all GBM cell lines and tumors and observed a positive correlation between intronic CpG methylation and *MGMT* expression and a negative correlation between exonic CpG methylation with *MGMT* expression. Overall, these studies described previously illustrated that there is great potential to use nCATS as a clinical tool for cancer precision medicine. The nCATS requires only ∼3 μg of genomic DNA and can target a large number of loci in a single reaction. The method will facilitate the use of nanopore sequencing in research and in the clinic and will be a very active area of development, and it is possible for us to see many new exciting applications and protocols in the future.

## Conclusion Remarks

In this review, we highlighted the advances of nanopore sequencing techniques for detecting methylation modifications and biological discoveries with their application in the context of cancer. The emergence of nanopore sequencing analysis has enabled direct methylation readout throughout genome, transcriptome, and target region sequences, including previously inaccessible epigenomic regions, such as highly repetitive areas. With respect to the epigenetic features on DNA, nanopore sequencing makes it possible to evaluate differential methylation and chromatin accessibility from a single high-throughput experiment, providing excellent opportunities to pinpoint cancer gene regulation. Moreover, the nanopore sequencing peculiarity of being able to directly sequence RNA with high accuracy allows a better study of differential modifications and expression from a single high-throughput reaction. Furthermore, the sequencing depth granted by nCATS is extremely useful for analyzing heterogeneous samples typically obtained from pre-clinical and clinical samples, shedding new light on the epigenetic changes in the cancer onset and progression. However, nanopore sequencing as a technology still under development and frequent updates in chemistry and software currently challenge its clinical application and need to be addressed to allow standardized diagnosis across laboratories. Further development and improvement of nanopore sequencing accuracy are also required to thoroughly resolve and decode cancer epigenetic changes at a single-base level.

## Author Contributions

SW and HL conceived the idea and revised the manuscript. JZ led the writing and produced the table and figure. SX wrote part of the manuscript. SW supervised the study and wrote the manuscript. All authors read and approved the final manuscript.

## Conflict of Interest

The authors declare that the research was conducted in the absence of any commercial or financial relationships that could be construed as a potential conflict of interest.
